# Esketamine combined with pregabalin on acute postoperative pain in patients undergoing resection of spinal neoplasms: study protocol for a randomized controlled trial

**DOI:** 10.1186/s13063-023-07178-3

**Published:** 2023-02-25

**Authors:** Wanchen Sun, Juan Wang, Jing Wang, Jingyi Fan, Yang Zhou, Yunzhen Wang, Ruquan Han

**Affiliations:** Department of Anesthesiology, Beijing Tiantan Hospital, Capital Medical University, No. 119, Southwest 4th Ring Road, Fengtai District, Beijing, People’s Republic of China 100070

**Keywords:** Esketamine, Pregabalin, Acute postoperative pain, Spinal neoplasms, Randomized controlled trial

## Abstract

**Background:**

Perioperative pain management is one of the most challenging issues for patients with spinal neoplasms. Inadequate postoperative analgesia usually leads to severe postsurgical pain, which could cause patients to suffer from many other related complications. Meanwhile, there is no appropriate analgesic strategy for patients with spinal neoplasms.

**Methods/design:**

This is a protocol for a randomized double-blind controlled trial to evaluate the effect of esketamine combined with pregabalin on postsurgical pain in spinal surgery. Patients aged 18 to 65 years scheduled for spinal neoplasm resection will be randomly allocated into the combined and control groups in a 1:1 ratio. In the combined group, esketamine will be given during the during the surgery procedure until 48-h postoperative period, and pregabalin will be taken from 2 h before the surgery to 2 weeks postoperatively. The control group will receive normal saline and placebo capsules at the same time points. Both groups received a background analgesic regimen by using patient-controlled intravenous analgesia (containing 100 μg sufentanil and 16 mg ondansetron) until 2 days after surgery. To ensure the accuracy and reliability of this trial, all the researchers and patients will be blinded until the completion of this study. The primary outcome will be the proportion of patients with acute moderate-to-severe postsurgical pain (visual analog scale, VAS ≥ 40, range: 0–100, with 0, no pain; 100, the worst pain) during the 48-h postoperative period. The secondary outcomes will include the maximal VAS scores (when the patients felt the most intense pain over the last 24 h before being interviewed) at 0–2 h, 2–24 h, 24–48 h, and 48–72 h after leaving the operating room and 24 h before discharge; the incidence of acute moderate-to-severe postsurgical pain at each other time point; chronic postsurgical pain assessment; neuropathic pain assessment; and the incidence of drug-related adverse events and other postoperative complications, such as postoperative delirium and postoperative nausea and vomiting (PONV).

**Discussion:**

The aim of this study was to evaluate the effect of esketamine combined with pregabalin on acute postsurgical pain in patients undergoing resection of spinal neoplasms. The safety of this perioperative pain management strategy will also be examined.

**Trial registration:**

ClinicalTrials.gov NCT05096468. Registered on October 27, 2021

**Supplementary Information:**

The online version contains supplementary material available at 10.1186/s13063-023-07178-3.

## Background

Pain is one of the most common complications for patients undergoing surgery. According to previous studies, almost 80% of patients undergoing spinal surgery suffer from acute postoperative pain [1], and over 60% of these patients might have persistent postoperative pain [2]. Poor perioperative pain management was an independent risk factor for persistent postoperative pain [3] and could increase the risk by 3 times [4]. Elderly patients with postoperative pain might have impaired cognitive function [5], which increases the risk of senile dementia [6]. Severe postoperative pain could also influence the emotional and mental health of patients [7, 8], which could be associated with unfavorable prognoses for the patients. Moreover, inadequate acute postsurgical pain management could result in longer hospitalization, decreased satisfaction, and higher hospital costs. Therefore, effective perioperative pain management, especially appropriate management of acute postoperative pain, is one of the most important links to help enhance the recovery of surgical patients.

One of the most challenging issues during the perioperative period is providing appropriate pain control measures to enhance the recovery of patients. Over the last decade, opioids have been the most commonly used drug in perioperative pain management strategies. However, the administration of opioids could be compounded by aspects of misuse, overdose, and addiction. Furthermore, clinical adverse events such as nausea and vomiting and respiratory depression have limited the use of opioids. In 2016, the American Society of Anesthesiologists (ASA) and American Society of Regional Anesthesia and Pain Medicine (ASRAPM) jointly published consensus guidelines for acute pain management. These guidelines indicate that ketamine [9] and pregabalin [10] can be used for the treatment of postsurgical pain.

The mechanisms underlying postsurgical pain might be peripheral and central sensitization because of nerve injury and inflammatory responses at the operative site. Previously, researchers have suggested that central pain sensitization might be related to N-methyl-d-aspartate (NMDA) receptor activation [11]. Ketamine, an NMDA receptor antagonist, reduces central pain sensitization by blocking the activity of NMDA receptors. A related meta-analysis found that intravenous ketamine could reduce VAS scores in the 24~48-h postoperative period and decrease opioid consumption [12]. S-Ketamine, one of the enantiomers of ketamine, is a more effective analgesic and has fewer side effects than ketamine. Regarding the effects of S-ketamine on acute postsurgical pain, a meta-analysis of 12 randomized controlled trials showed that intravenous S-ketamine could decrease the intensity of pain and opioid requirements only 24 h after surgery and may increase the incidence of psychotomimetic adverse events [13]. Unfortunately, the evidence level was only moderate or even low. Due to the differences in the S-ketamine intervention protocols and the types of surgery, more high-quality studies are needed.

At the same time, gabapentinoids (gabapentin and pregabalin) have gained more attention from researchers for their significant effect on neuralgia. These compounds can inhibit the modulation of neuronal excitability by blocking α2–δ subunits of presynaptic, voltage-dependent calcium channels, which are upregulated in central sensitization processes [11]. A meta-analysis of preoperative gabapentinoids for acute postoperative pain reported that gabapentinoids were associated with reduced pain scores and cumulative morphine consumption during the 48-h postoperative period [1]. In the same year, another meta-analysis showed the effectiveness of preoperative pregabalin on acute postsurgical pain [14]. In contrast, in 2020, a meta-analysis including 281 trials investigated the effect of perioperative gabapentinoids on postoperative acute pain and reported the noneffective analgesic effects of gabapentinoids in adult patients during the postoperative period [15].

Thus, we designed a randomized controlled study to explore the analgesic effects of a combination of esketamine and pregabalin after resection of spinal neoplasms to investigate whether the combined therapy could improve postoperative analgesia. The fundamental aim of this study was to find an effective and safe analgesic treatment to help patients undergoing spinal surgery and provide a new perspective regarding perioperative pain management strategies.

## Methods/design

### Study design

This study will be a single-center, randomized, double-blind, controlled trial with two parallel study arms. This equivalence trial aims to evaluate the analgesic effects of esketamine in combination with pregabalin after resection of spinal neoplasms. The enrolled subjects will be randomly allocated into the combined group (esketamine and pregabalin) and the control group (normal saline and placebo) in a 1:1 ratio. This study was approved by the Institutional Review Board of Beijing Tiantan Hospital, Capital Medical University, Beijing, China (KY-2021-081-02). This study (protocol version 1.4/date 2021.10.08) was registered on ClinicalTrials.gov on October 27, 2021 (NCT05096468). All participants or their legal representatives will provide written informed consent after screening and before randomization.

### Study setting and recruitment procedure

**Fig. 1:**
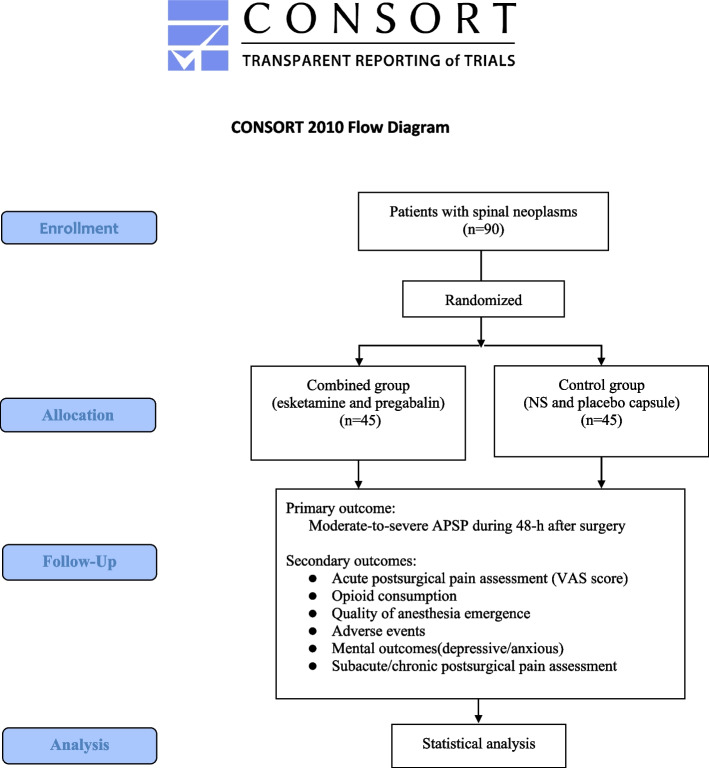
Participant timeline showing the patient contacts in the study: enrolment, randomization, intervention, and follow-up

The recruitment of this trial will be conducted in Beijing Tiantan Hospital from 1 December 2021 to 31 August 2022, and the follow-up will be until 31 December 2022. Based on the electronic medical records of the hospital, potential participants will be individually screened after admission. Almost 100 patients underwent surgery in the spinal neurosurgery department at Beijing Tiantan Hospital in 1 month, and the proportion of patients with spinal neoplasms was nearly 60% in this population. The sample size is only 90 for our study. Therefore, the predicted 8-month duration of this study is enough to complete the recruitment. No extra measures will be taken to recruit a sufficient number. Participants meeting the eligibility criteria will be visited by the research staff to sign informed consent forms and provide baseline information. The flow chart of the study is shown in Fig. 1.

### Study population

Patients meeting the following inclusion criteria and not meeting the exclusion criteria at the time of randomization will be considered for enrollment.

#### Inclusion criteria:

1. Scheduled to undergo elective resection of spinal neoplasms
2. Age between 18 and 65 years old
3. ASA I–III
4. Signed informed consent

#### Exclusion criteria:

1. Previous adverse reaction to ketamine, S-ketamine, or pregabalin
2. A diagnosed history of severe chronic pain (defined as pain persisting at least 3 months before the surgery for any reason, with an NRS score greater than 6)
3. Long-term medical history of analgesic treatment (gabapentin/opioids/ketamine)
4. Aphasia or inability to cooperate with the pain assessments
5. A diagnosed history of hepatic and renal dysfunction
6. A diagnosed history of psychiatric disorder
7. The patient was treated with gabapentin/pregabalin in the last 3 months
8. Drug use disorder
9. Body mass index > 35 kg/m^2^
10. Pregnancy or lactation

### Randomization and blinding

Patients will be randomized 1 day before surgery using blocked, stratified randomization by independent research staff to 1 of 2 treatment groups (the combined group or the control group) after study enrollment. We will randomize the patients in a 1:1 ratio in blocks of four. The randomized allocation list will be generated by using a real-time, online system on the day before surgery. The randomization results will be maintained by independent researchers who will not participate in any other work of this study to guarantee allocation blinding. The presence of moderate or severe preoperative pain will be used as a basis for the stratification of patients (evaluated by a visual analog scale, range: 0–100, with 0, no pain; 100, the worst pain; moderate or severe preoperative pain is defined by a VAS score equal to or more than 40 mm).

Allocation concealment will be achieved using study capsules and intravenous infusions with identical appearance for placebo and drugs. All the trial drugs will be provided by a medicine company (Hengrui Induction, Jiangsu, China) with no other contribution to this trial. The appearance of all trial capsules (placebo capsules containing starch and pregabalin capsules) will be the same. Esketamine will be diluted to a concentration of 0.5 mg/ml with normal saline. Colorless 50-ml syringes containing clear esketamine or placebo (normal saline) solution will be prepared in a blinded manner by an independent research assistant. The randomization code will be marked on the syringes and the drug bottles of the study capsules. Only the study investigator who assigns participants to interventions will be unblinded to the treatment assignment. All other study researchers will be blinded to the treatment assignment, including responsible anesthesiologists and the neurosurgeons in charge of the patients, the outcome assessors, data analysts, and other relevant investigators.

### Intervention

In the combined group, the patients will be given both esketamine and pregabalin during the perioperative period. The patients will receive 2 capsules of pregabalin (75 mg/capsule, 150 mg total) 1~2 h before the surgery and 1 capsule twice daily during the 7-day postoperative period (75 mg/capsule, BID; 150 mg total; POD 1~7), followed by dose reduction to 1 capsule once daily for 7 days (75 mg/capsule, QD; 75 mg total; POD 8~14). At the same time, patients will be administered a bolus dose of 0.5 mg/kg esketamine after the induction of anesthesia and then receive continuous intravenous infusion at 0.12 mg/kg ·h (2 μg/kg ·min) during the 48-h postoperative period.

In the control group, the patients will be given the same number of placebo capsules as the combined group at the same time points (two placebo capsules 1~2 h preoperatively and twice daily postoperatively for 7 days, followed by a reduction to a single capsule once daily for 7 days), alongside a normal saline bolus after induction of anesthesia and intravenous saline infusion for the 48-h postoperative period.

### Perioperative anesthesia management

Perioperative anesthesia management will be standardized. After entering the operating room, the patients will be given standard electrocardiography monitoring. Anesthesia will be induced with propofol, sufentanil, and rocuronium/cisatracurium. Total intravenous anesthesia depends on target-controlled infusions and will be chosen to maintain anesthesia with propofol and sufentanil and keep the bispectral index between 40 and 60. Remifentanil will be prohibited in this study. For the potential impact on the evaluation of the operation, nerve block will not be considered. In addition, local wound infiltration (0.5% ropivacaine 20 ml) will be permitted before skin suturing if required by the surgeon. All the participants will be given the usual postoperative analgesia therapy by postoperative patient-controlled intravenous analgesia (PCIA, 0.5 ml per bolus (containing 0.5 μg sufentanil) with a lockout period of 15 min, 100 ml total), which will include 100 μg sufentanil and 16 mg ondansetron during the 2-day postoperative period. In the ward, all patients will receive parecoxib sodium (40 mg IV BID) or flurbiprofen axetil (50 mg IV BID) to receive the standard postoperative analgesic for at least 3 days. If the patients are experiencing moderate or severe pain (VAS score ≥ 40 mm), rescue analgesia will be given, including 0.5 μg sufentanil IV in the postanesthesia care unit (PACU) or 1 tablet Tylox (contained 325 mg acetaminophen and 5 mg oxycodone per tablet) in the ward. If the pain persists, the patients will receive 1 dose of 50 to 100 mg of intramuscular tramadol. All analgesic doses and names will be clearly recorded.

### Provisions for posttrial care

All of the participants will receive conventional postoperative care in the ward. The postoperative treatment will not be changed whether the patients enter the trial. The interventions of this trial are a combination of analgesic management added to the basic analgesic treatment, which will help the patients to get more comfortable feelings. The interventions are common drugs in the clinic, and the dosages are lower. Thus, the patients will be at low risk during the interventional period. Therefore, there is no anticipated harm or special posttrial care for the participants.

### Outcomes

The assessment of outcomes will be performed by two independent researchers. The results during hospitalization will be collected by the investigators through bedside interviews. The collection of postdischarge outcomes will be performed by telephone interview.

**Fig. 2:**
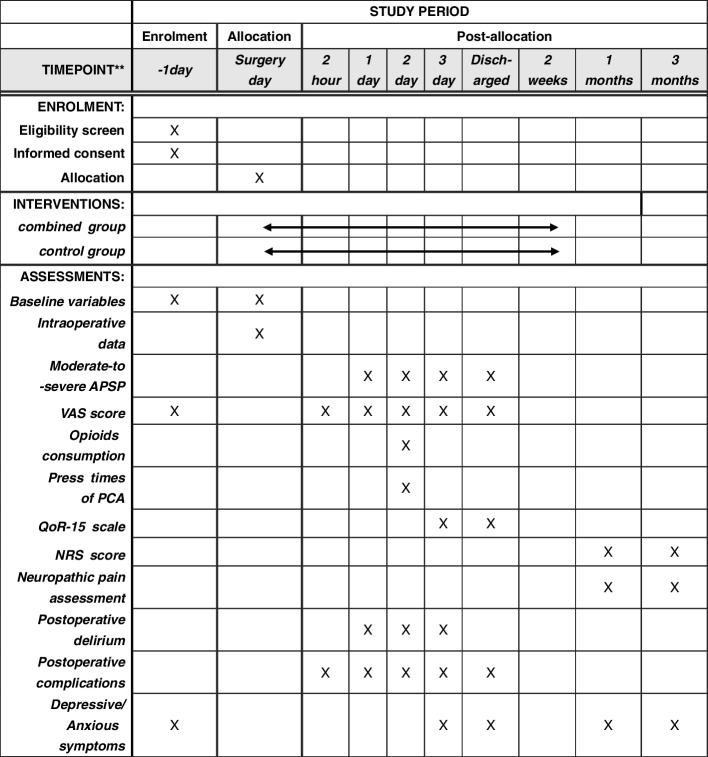
Standard Protocol Items: Recommendations for Interventional Trials (SPIRIT) figure showing the schedule of enrollment, intervention, and assessments

The primary outcome was the proportion of patients with acute moderate-to-severe postsurgical pain during the 48-h postoperative period (defined as a VAS score ≥ 40 mm). The schedule of enrollment, intervention, and assessments is presented in Fig. 2.

Secondary outcomes include the following:

1. Acute pain assessment: the maximal VAS score (when the patients felt the most intense pain over the last 24 h before being interviewed) 0–2 h, 2–24 h, 24–48 h, 48–72 h after leaving the operating room and 24 h before discharge; the incidence of acute moderate-to-severe postsurgical pain at each other time point (defined as the VAS score ≥ 40 mm)
2. Subacute or chronic pain assessment: The incidence of postsurgical pain was assessed by a number rating scale (NRS: 0~10) at 1 month and 3 months (defined as an NRS score > 3)
3. Neuropathic pain assessment: the short-form McGill pain questionnaire-2 (SF-MPQ-2) scores at 1 month and 3 months postoperatively
4. Total opioid consumption within the 48 h period after surgery
5. Effective or noneffective button presses for PCIA
6. Quality of anesthesia emergence evaluated by a 15-item quality of recovery scale (QoR-15) on POD3 and at discharge
7. The incidence of psychotic side effects (such as hallucinations and dissociative symptoms) was determined using the Brief Psychiatric Rating Scale (BPRS) and Clinician-Administered Dissociative States Scale (CADSS) at 2 h after surgery, POD1, POD2, and POD3
8. Adverse events included drug-related (palpitation, hypertension, dizziness, diplopia) and operation- or anesthesia-related complications, the incidence of PONV, and the incidence of postsurgical delirium at 2 h after surgery, POD1, POD2, and POD3
9. Other outcomes: perioperative depressive/anxious symptoms assessed by the Patient Health Questionnaire 9 (PHQ-9) and Generalized Anxiety Disorder scale (GAD-7) on POD3, at discharge, and 1 month and 3 months after surgery

### Data management

All the clinical information will be recorded in the unified case report forms designed specifically for this project and other paper documents, including informed consent forms and the protocol. After the follow-up is completed, the research paper documents will be locked in a single secure office in Beijing Tiantan Hospital, and the key will be kept by only the primary investigator and trial manager. Research data will be coded with a unique trial ID number, and no personal identifiers will be contained in the research data. The trial ID number that links identifying information to study subjects will be accessible only to the study investigators. The data will be entered into an enciphered electronic database after completion of this study. Only the principal investigator will know the password of this electronic database. All of the paper materials and the electronic database will be managed by the principal investigator until the study is complete.

### Statistical analysis

Continuous data will be described as the mean with standard deviation (SD) or median with interquartile range (IQR) based on its distribution. The histogram and Kolmogorov–Smirnov tests will be used to evaluate the normality of the data distribution. Categorical variables will be summarized as counts and percentages. The primary outcome is categorical and will be analyzed by using the chi-square test or Fisher’s exact test. Regarding the secondary outcomes, based on the type of each variable, the difference between groups will be analyzed using *t* tests, Mann–Whitney *U* tests, and chi-square or Fisher’s exact tests as appropriate. We will use the modified intension-to-treat (mITT) analysis to report our results, and we will use per-protocol (PP) analysis to perform the sensitivity analysis. The methods for estimating effects and 95% CIs will be reported in our outcomes. Sensitivity analyses will be applied to determine the statistical nature of the missing data. A two-sided *P* value of 0.05 was considered statistically significant for the primary outcome. Because of the repeated measurement, the Holm–Bonferroni method will be used to adjust the *P* value for secondary outcomes. Statistical analysis will be implemented using SPSS version 23.0 software and Stata version 14.0.

### Sample size calculation

According to a previous randomized controlled trial that investigated the effect of combined ketamine and pregabalin on persistent postoperative pain, the prevalence of moderate-to-severe acute postoperative pain at 24 h after surgery was 42% in the combined group and 78% in the control group [16]. Among patients with preoperative pain, the incidence of acute moderate-to-severe postsurgical pain in the population undergoing spinal surgery is nearly 80% [1]. Based on the above data and the current clinical situation, we estimate that the incidence of acute moderate-to-severe postsurgical pain will be 30% in the combined group and 60% in the control group, with a total 2-sided type I error rate of 0.05 and a 20% withdrawal rate. Ninety patients will have to be enrolled to achieve a statistical power of 80% (*P* values of 0.05).

### Interim analyses

Because of the low-risk interventions, the short-term duration, and the small sample size, there are no interim analyses planned.

### Oversight and monitoring

#### Composition of the coordinating center and trial steering committee

This is a single-site study designed, performed, and coordinated at Beijing Tiantan Hospital. There is no steering committee or adjudication committee. Day-to-day running and management of the trial are based on the regular meeting and led by the chief investigator (CI, Ruquan Han). The study team meets via a teleconference or face-to-face at least twice a month. The content of regular meetings includes the following: (1) study investigators report the process of the trial, including recruitment, informed consent, assessment, and intervention procedures according to protocol; (2) trial managers raise the problems during the performance of the study; and (3) principal investigators provide oversight for all aspects of the trial and give support to study investigators in each link.

#### Composition of the data monitoring committee, its role, and reporting structure

In agreement with the Institutional Review Board (IRB) of Beijing Tiantan Hospital, a DSMB was not appointed for this study. Because this study is a short-term study, the interventional drugs of this trial are conventional clinical analgesics with safe dosages. The risks of the interventions are minimal. Moreover, enrolled subjects in this study are adjunctive to routine treatment through the spinal neurosurgery department in Beijing Tiantan Hospital. The population in this trial will be monitored carefully during hospitalization, and regular perioperative treatment will not be altered by study participation. Two knowledgeable anesthesiologists and a spinal neurosurgeon were responsible for the safety of this study. Once adverse events are suspected, they will be asked to diagnose whether the suspected adverse event is related to interventions and will guide the next treatments for safety measures when indicated. The study team will submit the annual tracking reports to IRB institutions to upload the process information of the study once a year. If there is any modification in this trial, the trial manager will need to submit the application in a timely manner, and the IRB will approve if it is feasible.

#### Adverse event reporting and harms

The follow-up investigators will pay close attention to the subjects during the intervention. If any adverse events (AEs) occur, the responsible investigator will report to the principal researcher immediately. If AEs are common and the patients’ symptoms are mild, we will supply thorough symptomatic support and treatments with close monitoring. If AEs become severe, the intervention will be stopped at once, professional doctors from various fields to immediately help the patients. Based on the relationships between AEs or serious adverse events (SAEs) and the drugs being assessed in this study, the project management team will consider whether unblinded allocated interventions are needed. If the AEs are so serious that they might impact regular clinical treatment and threaten patient safety, the research team will perform the emergency unblinding process and report the interventions to the surgeon in charge of the subjects at once. All AEs will be followed up until resolution. All adverse events will be documented in the final written report of this study and will be reported to the Institutional Review Board by the principal investigator.

### Patient and public involvement

Patients and the public were not involved in the trial design. Participants will have access to the findings of the study on request.

## Discussion

This proposed study will measure the efficacy and safety of esketamine combined with pregabalin on postsurgical pain in patients undergoing elective resection of spinal neoplasms. The participants will receive esketamine from the beginning of surgery until 48 h postoperation, along with a total dose of 150 mg pregabalin for 1 week postoperatively (POD 1~7, including the operation day) followed by a reduced dose of 75 mg pregabalin for the next week (POD 8~14). Both acute and chronic postoperative pain will be assessed during the 3 months after surgery. Other complications and safety outcomes will also be observed.

The primary concern is the potential inadequate analgesia in the control group after the surgery. The PCIA device is not a regular part of the therapeutic regimen during the postoperative period in this study hospital. As a result, we designed a usual PCIA procedure for all the participants to ensure basic postoperative analgesic treatment. If the participants experienced a moderate or severe level of pain, rescue analgesia was administered. The secondary concern is in regard to active placebo for the preoperative pregabalin capsule. In 2017, Hah’s team designed a randomized clinical trial to evaluate the effects of perioperative gabapentin on pain resolution in a mixed surgical population [17]. The researchers considered the sedating effects of preoperative gabapentin, so they chose lorazepam as the preoperative active placebo. Sleepiness is one of the common side effects of this kind of drug. However, in our pilot study, we did not observe obvious sleepiness in the participants before the surgery after taking pregabalin, and we will administer an injection of midazolam as a standard step after the patients enter the operating room; therefore, our research team decided to use the starch capsule as the placebo capsule at all time points. Furthermore, we chose total intravenous anesthesia as the standard maintenance of anesthesia with propofol and sufentanil, and propofol injection depended on target-controlled infusion (TCI). Our anesthesia management protocols might be different from those of previous studies. However, total intravenous anesthesia dependent on TCI technology can be more convenient to calculate and analyze possible drug interactions after the completion of the study. Moreover, we did not previously find any adverse events during the pilot study that are of concern, such as intraoperative awareness or delayed recovery.

The choice of the dosages of these two trial drugs was deliberate. Based on a related meta-analysis [3], intravenous ketamine does not increase complications related to the central nervous system (such as delirium and hallucinations). A meta-analysis [18] on the effect of pregabalin on chronic pain suggested that doses as high as 300~600 mg pregabalin per day do not increase the incidence of severe adverse events. Moreover, the guidelines on pain management [10] have suggested the following recommended dosages of these two drugs: a 0.5-mg/kg bolus of ketamine with a continuous intravenous infusion dose of 0.12 mg/kg h (2 μg/kg min) and a maximum continuous infusion dose that does not go over 10 μg/kg min; a 150~300-mg preoperative dose of pregabalin and a postoperative dose of 150~300 mg per day. Based on the above information, these two trial drugs in our study will be used at lower dosages. The purpose of this dose design is to keep the participants as safe as possible while relieving postsurgical pain.

In conclusion, this study is a randomized controlled trial to explore a better method to treat postsurgical pain. The expected outcome is that perioperative esketamine combined with pregabalin can safely and effectively relieve postsurgical pain, and the results of this study can expand the understanding of perioperative pain management.

### Trial status

This study was approved by the Institutional Review Board of Beijing Tiantan Hospital, Capital Medical University, Beijing, China, on November 20, 2021 (KY-2021-081-02). It was registered on ClinicalTrials.gov on October 27, 2021 (NCT05096468). The first participant will be recruited on December 1, 2021. The conclusions of this study will be published in peer-reviewed journals.

## Supplementary Information

**Additional file 1.** CONSORT 2010 Flow Diagram.

## Data Availability

The datasets analyzed during the current study and statistical code will be available from the primary investigator (Ruquan Han, Email: ruquan.han@ccmu.edu.cn) upon reasonable request after the publication of the study results.

## Abbreviations

VAS: Visual analog scale
PONV: Postoperative nausea and vomiting
ASA: American Society of Anesthesiologists
ASRAPM: American Society of Regional Anesthesia and Pain Medicine
NMDA: N-Methyl-d-aspartate
POD: Postoperative day
NRS: Number rating scale
SF-MPQ-2: The short-form McGill pain questionnaire-2
PCIA: Postoperative patient-controlled intravenous analgesia
QoR-15: 15-Item quality of recovery scale
BPRS: Brief Psychiatric Rating Scale
CADSS: Clinician-Administered Dissociative States Scale
PHQ-9: Patient Health Questionnaire 9
GAD-7: Generalized Anxiety Disorder scale 7
SD: Standard deviation
IQR: Interquartile range
AEs: Adverse events
SAEs: Serious adverse events
TCI: Target-controlled infusion
