# Supplementary material for: Esketamine combined with pregabalin on acute postoperative pain in patients undergoing resection of spinal neoplasms: study protocol for a randomized controlled trial
Source: Trials. 2023 Feb 25;24:144. doi: 10.1186/s13063-023-07178-3 (PMC9960454; doi:10.1186/s13063-023-07178-3)
Supplement: Supplementary file 1 — Additional file 1. CONSORT 2010 Flow Diagram. [file 13063_2023_7178_MOESM1_ESM.doc]

**
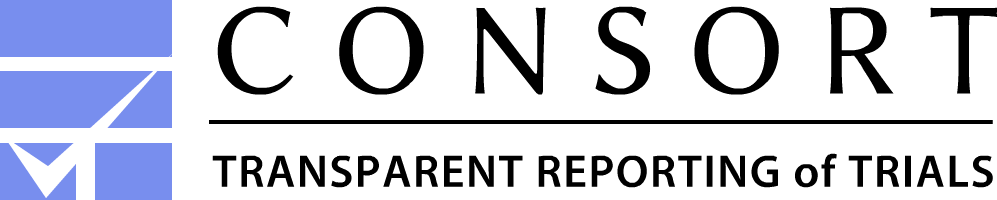
**

**CONSORT 2010 Flow Diagram**

**Allocation**

**Analysis**

**Follow-Up**

**Enrollment**

Patients with spinal neoplasms (n=90)

Primary outcome:

Moderate-to-severe APSP during 48-h after surgery

Secondary outcomes:

- Acute postsurgical pain assessment (VAS score)
- Opioid consumption
- Quality of anesthesia emergence
- Adverse events
- Mental outcomes(depressive/anxious)
- Subacute/chronic postsurgical pain assessment

Combined group

(esketamine and pregabalin)

(n=45)

Control group

(NS and placebo capsule)

(n=45)

Statistical analysis

Randomized
